# Outcomes of Tricuspid Transcatheter Edge-to-Edge Valve Repair in End-Stage Renal Disease

**DOI:** 10.1016/j.jscai.2025.102643

**Published:** 2025-06-17

**Authors:** Lamiya Rodriguez, Keniel Pierre, JoAnn Lindenfeld, Rebecca Hung, Tarek Absi, Angela Lowenstern, Brian Lindman, Colin Barker, Kashish Goel

**Affiliations:** Division of Cardiology, Vanderbilt University Medical Center, Nashville, Tennessee

**Keywords:** transcatheter edge-to-edge repair, TriClip, tricuspid regurgitation

## Introduction

Patients with end-stage renal disease (ESRD) experience progressive tricuspid regurgitation (TR) due to chronic volume overload. Severe TR leads to worsening symptoms and reduced efficacy of hemodialysis due to reduced stroke volume. Patients with ESRD are at high risk for complications and a poor outcome after surgical valve repair. Although tricuspid transcatheter edge-to-edge repair (T-TEER) provides a promising alternative for those who are not good candidates for cardiac surgery, its effect on patients with ESRD remains unexplored as these patients have historically been excluded from clinical trials. This study’s objective was to evaluate the outcomes of T-TEER in patients with ESRD.

## Methods

We retrospectively reviewed ESRD patients who underwent T-TEER using the MitraClip system (Abbott) at Vanderbilt University Medical Center. The institutional database was queried to identify all patients who underwent off-label T-TEER using a MitraClip device from 2019 to 2023. Of the 61 patients who underwent T-TEER, 6 had ESRD. ESRD was defined as requiring permanent dialysis as an outpatient. All patients received hemodialysis through a fistula. The indication for T-TEER was severe functional TR causing symptomatic fluid overload and inability to tolerate hemodialysis. Baseline TR grade was determined based on the transesophageal echocardiogram prior to intervention (Table 1). Discharge and follow-up TR grades were obtained from transthoracic echocardiograms. All the echocardiograms were read by level III echocardiographers for clinical purposes using either quantitative or qualitative analysis. All patients were considered high risk for cardiac surgery and thereby underwent T-TEER after heart team discussions. The procedure was performed under general anesthesia via the right femoral vein. The following outcomes were assessed at discharge, 30 days, and 1 year: change in TR grade, New York Heart Association (NYHA) class, and vital status. Changes in TR grade were evaluated using transthoracic echocardiograms. Kansas City Cardiomyopathy Questionnaire scores were obtained prior to intervention, with the majority of scores reporting poor health status (Table 1). However, given the retrospective nature of this study, we were unable to obtain follow-up Kansas City Cardiomyopathy Questionnaire scores. Device success and procedural complications including vascular, nonvascular, and cardiac complications were defined using the Tricuspid Valve Academic Research Consortium guidelines.^1^ Improvement in dialysis tolerability was defined as the ability to have additional fluid removed during dialysis without symptomatic hypotension. This was reported by the patients during follow-up and documented by their physician. We were unable to objectively measure dialysis tolerability due to lack of access to dialysis center reports.

**Table 1:** Baseline, procedural, and follow-up characteristics.

	Case 1	Case 2	Case 3	Case 4	Case 5	Case 6
Age, y	63	72	83	58	43	68
Sex	Male	Female	Male	Female	Female	Male
Years since starting dialysis	6	3	2	6	1	8
Coronary artery disease	Yes	Yes	Yes	No	Yes	Yes
Atrial fibrillation	No	Yes	Yes	No	No	Yes
Hypertension	Yes	No	Yes	Yes	Yes	No
Diabetes	Yes	No	Yes	No	Yes	No
NYHA class	3	4	3	3	3	3
KCCQ-SS	12	19	40	57	29	–
BNP, pg/mL	9330	3483	621	1500	105	2845
Etiology of TR	Functional (mixed)	Functional (atrial)	Functional (atrial)	Functional (atrial)	Functional (atrial)	Functional (atrial)
TEE findings						
TR severity	Severe	Torrential	Massive	Massive	Severe	Torrential
LVEF, %	50	60	60	60	60	25
RV dysfunction	None	Moderate	Mild	None	Mild	Mild
RV dilation	Mild	Moderate	Moderate	Moderate	Normal	Normal
RA dilation	Mild	Severe	Severe	Severe	Mild	Severe
RVSP, mm Hg	46	50	64	49	42	50
T-TEER procedural characteristics						
TEER device	MitraClip	MitraClip	MitraClip	MitraClip	MitraClip	MitraClip
No. of clips	1 (XTW)	3 (2 XTW, XT)	2 (XTW)	1 (XTW)	1 (XTW)	2 (XTW)
Residual TR, grade	Mild	Moderate	Mild	Mild	Mild	Mild
TR grade reduction	2	3	3	3	2	4
Complications^a^	None	None	None	None	None	None
TTE findings, 30 d						
TR severity	Moderate	Severe	Mild	Severe	Mild	Moderate
RV dysfunction	Mild	Moderate	Mild	None	None	Mild
RV dilation	Moderate	Severe	Moderate	Moderate	Normal	Normal
NYHA class, 30 d	4	3	1	1	1	2
Follow-up, 12 mo						
TR severity (TTE)	–	–	Mild	Moderate	Mild	Moderate
RV dysfunction (TTE)	–	–	Mild	None	None	None
RV dilation (TTE)	–	–	Moderate	Moderate	Normal	Normal
NYHA class	–	–	1	1	-	2
Death	No	No	No	No	No	No
Reintervention	No	No	No	No	No	No
Device embolization	No	No	No	No	No	No

BNP, B-type natriuretic peptide; KCCQ-SS, Kansas City Cardiomyopathy Questionnaire summary score; LVEF, left ventricular ejection fraction; NYHA, New York Heart Association; RA, right atrium; RV right ventricle; RVSP, right ventricular systolic pressure; TR, tricuspid regurgitation; TEE, transesophageal echocardiogram; TTE, transthoracic echocardiogram; T-TEER, tricuspid transcatheter edge-to-edge repair.

a Vascular, nonvascular access, and cardiac complications as defined by Tricuspid Vale Academic Research Consortium guidelines.

## Results

Median age was 66 years (range, 43-83 years), and 50% were women. Baseline characteristics, comorbidities, echocardiographic and hemodynamic data, and procedural outcomes are presented in Table 1. At baseline, 33.3% had severe TR and 66.6% had massive or torrential TR. At baseline, all patients had secondary TR. The etiology of secondary TR was predominantly atrial (4 of 6 patients had severe right atrial dilation) (Table 1). All patients underwent a successful procedure with no complications. The median number of clips per case was 1.5, with 83% being XTW. All patients achieved at least a 2-grade reduction in TR after T-TEER. Specifically, 5 patients had mild TR and 1 had moderate TR at discharge. Mean right atrial pressure (pre, 18.5 mm Hg; post, 13.3 mm Hg) and V wave (pre, 28.5 mm Hg; post, 18.6 mm Hg) were lower after T-TEER (n = 4).

At the 30-day follow-up, all patients were alive. Of the 6 patients, 4 had less than or equal to moderate TR, 2 had severe TR (Supplemental Figure S1). All patients except one had improvement in NYHA class (Table 1). During follow-up, hemodialysis tolerability improved in 50% of patients. At 1 year, 4 patients had complete follow-up. Among these patients, all had less than or equal to moderate TR at 1 year (Supplemental Figure S1) and noted improvement in NYHA class. From discharge to 1-year follow-up, there were no procedural complications, single leaflet detachment, major device complications, concomitant procedures, or significant adverse events, including death, heart failure hospitalizations, stroke, major bleeding, or the need for tricuspid valve reintervention.

## Discussion

In this small series of ESRD patients undergoing T-TEER, we found that the procedure was safe and effective. T-TEER led to successful reduction in TR at 30 days and 1 year and was associated with improvement in NYHA class and dialysis tolerability. There were no procedural complications, and all patients were alive at 1 year.

TR is common in patients with ESRD, with up to 15% having moderate to severe TR.^2^ Management includes a trial of extra fluid removal during dialysis sessions; however, this can lead to hypotension. Less fluid removal leads to volume overload and shortness of breath. Given their comorbidities, patients with ESRD are frequently considered high risk for traditional tricuspid valve surgery, which has a high mortality rate.^3^ Although transcatheter tricuspid valve interventions have been approved recently, ESRD patients were excluded from these clinical trials.^4^ Thus, there are no data on the outcome of tricuspid interventions in ESRD. Although this is a small study, it is the first to report outcomes of T-TEER in ESRD patients.

We found that T-TEER was associated with at least a 2-grade reduction in TR severity in all patients at discharge and 66% of patients at 30 days. It is possible that outcomes will be better with a dedicated TriClip system (Abbott), which is now approved for commercial use. Another important finding of the study was improved hemodialysis tolerability in 50% of the patients. This is a crucial factor in patients with ESRD and severe TR, and our study suggests that treating TR may have this additional benefit. Furthermore, many of these patients may be candidates for renal transplant, and transcatheter options to treat TR may help their qualification. Prospective evaluation of these findings in future studies, including use of the on-label TriClip system, is required.

There were no procedural complications such as single leaflet device attachment, and all patients were discharged alive. In addition, all patients were alive during follow-up. If this finding can be replicated in larger studies, future TR trials may consider including ESRD patients. This study has several limitations. This is a retrospective study performed at a single institution and with a small cohort. As such, no major conclusions can be drawn, and the results are only hypothesis-generating. All the cases were performed using the off-label MitraClip system, and thus the results may not be applicable to the TriClip system.

## Conclusion

This small retrospective study provides evidence that T-TEER is effective in ESRD patients and results in significant clinical benefits, including enhanced hemodialysis tolerance and symptomatic relief. Prospective multicenter studies using the approved TriClip system could provide additional information in patients with ESRD.

## Supplementary material

Supplemental Figure S1 Residual Tricuspid Regurgitation and Magnitude of Reduction of Tricuspid Regurgitation at 30 Days and 1 Year Compared to Pre-Procedure.

## Footnotes

To access the supplementary material accompanying this article, visit the online version of the *Journal of the Society for Cardiovascular Angiography & Interventions* at 10.1016/j.jscai.2025.102643
