# Supplementary figures and images for: Outcomes of Tricuspid Transcatheter Edge-to-Edge Valve Repair in End-Stage Renal Disease
Source: J Soc Cardiovasc Angiogr Interv. 2025 Jun 17;4(6):102643. doi: 10.1016/j.jscai.2025.102643 (PMC12230442; doi:10.1016/j.jscai.2025.102643)

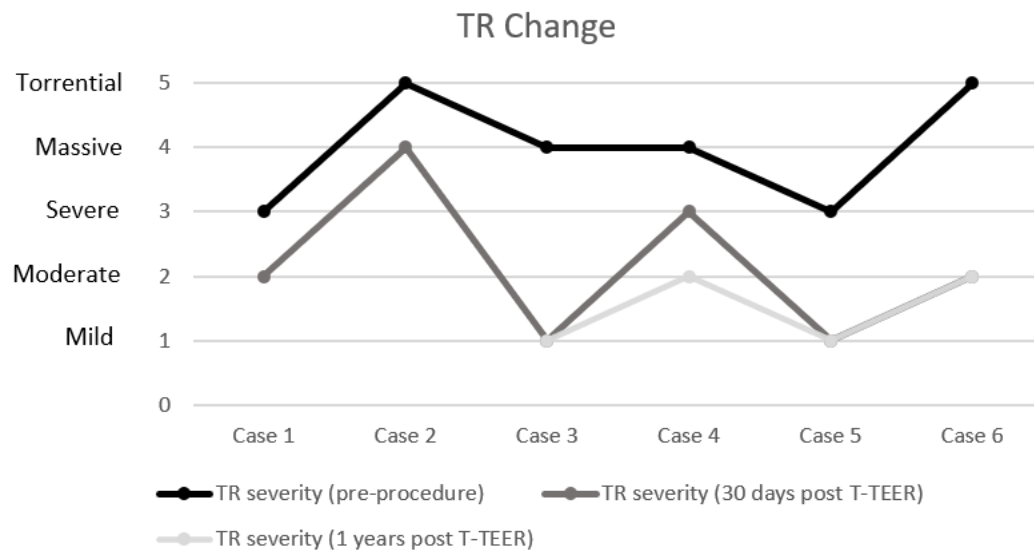

Supplement: Supplemental Figure S1 — Residual Tricuspid Regurgitation and Magnitude of Reduction of Tricuspid Regurgitation at 30 Days and 1 Year Compared to Pre-Procedure. [file mmc1.pdf]
